# Investigation of pre-hospital emergency missions in Mazandaran following jet ski and ATV accidents on the provincial coastline from 2020 to 2023

**Published:** 2024-10

**Authors:** Yahya Saleh Tabari, Zoya Hadinejad, Zeinab Sajadi, Mohammad Shadman, Ebrahim Zare, Hassan Talebi Ghadicolaei

**Affiliations:** ^*a*^ School of Medicine, Pharmaceutical Sciences Research Center, Hemoglobinopathy Institute, Mazandaran University of Medical Sciences, Sari, Iran.; ^*b*^ School of Allied Medical Sciences, Mazandaran University of Medical Sciences, Sari, Iran.; ^*c*^ Emergency Medical Services, Mazandaran University of Medical Sciences, Sari, Iran.; ^*d*^ Emergency Operation Center, Ministry of Health and Medical Education, Tehran, Iran.

## Abstract

**Background:**

Drowning and its related complications claim numerous lives worldwide annually. In recent years, in Mazandaran, the use of jet skis and All-Terrain Vehicles (ATVs) along the province's coastlines has led to injuries, drownings, and fatalities, creating a new type of incident. This study aimed to investigate the epidemiology of drowning and injuries resulting from jet ski and ATV accidents.

**Methods:**

This retrospective cross-sectional study utilized data from mission forms of Emergency Medical Services in Mazandaran from 20 March 2020 to the end of 20 March 2024. Data such as age, sex, accident location, native or non-native status, mission outcome (transferred to the medical center, canceled mission, outpatient treatment, death, etc.), and time and date of the incident were extracted. Data analysis was performed using SPSS version 19 and the chi-square test.

**Results:**

In the desired period, 404 cases were recorded in EMS. Among ATV accidents, 168 cases (46.15%) were male and 196 (53.85% ) were female. The average age of the victims was 35 years. Most victims (n=280, 69.3% ) were transferred to medical centers, 49 (12.2%) received outpatient treatment on-site, and 34 (8.4%) refused transfer. Ten percent of the missions were canceled. One 33-year-old man died from trauma caused by an ATV accident on the Mahmoodabad beach before the ambulance arrived. In cases of jet ski accidents, out of the 7 cases recorded over the 4-year study period, 5 were male and 4 female. Four victims were transferred to medical centers, and 5 received on-site treatment.

**Conclusions:**

The number of traumas and drownings following jet ski and ATV accidents has increased, posing a new threat to the health of tourists and locals. Recommendations such as wearing helmets and life jackets, observing safe driving speeds on ATVs, and using these recreational vehicles on secluded beaches and designated areas can effectively reduce the number of victims.

**Keywords:**

Epidemiology, Trauma, Drowning, Motor vehicles

